# Generation of Monkey Induced Pluripotent Stem Cell-Derived Cartilage Lacking Major Histocompatibility Complex Class I Molecules on the Cell Surface

**DOI:** 10.1089/ten.tea.2021.0053

**Published:** 2022-01-17

**Authors:** Yuki Okutani, Kengo Abe, Akihiro Yamashita, Miho Morioka, Shuichi Matsuda, Noriyuki Tsumaki

**Affiliations:** ^1^ Cell Induction and Regulation Field, Department of Clinical Application, Center for iPS Cell Research and Application, Kyoto University, Kyoto, Japan.; ^2^ Department of Orthopaedic Surgery, Graduate School of Medicine, Kyoto University, Kyoto, Japan.

**Keywords:** cartilage, chondrocytes, iPS cells, allogeneic transplantation, MHC, monkey

## Abstract

Due to the poor capacity for articular cartilage to regenerate, its damage tends to result in progressively degenerating conditions such as osteoarthritis. To repair the damage, the transplantation of allogeneic human induced pluripotent stem cell (iPSC)-derived cartilage is being considered. However, although allogeneic cartilage transplantation is effective, immunological reactions can occur. One hypothetical solution is to delete the expression of major histocompatibility complex (MHC) class I molecules to reduce the immunological reactions. For this purpose, we deleted the β2 microglobulin (B2M) gene in a cynomolgus monkey (crab-eating monkey [Macaca fascicularis]) iPS cells (cyiPSCs) to obtain *B2M^−/−^* cyiPSCs using the CRISPR/Cas9 system. Western blot analysis confirmed *B2M^−/−^* cyiPSCs lacked B2M protein, which is necessary for MHC class I molecules to be transported to and expressed on the cell surface by forming multimers with B2M. Flow cytometry analysis revealed no *B2M^−/−^* cyiPSCs expressed MHC class I molecules on their surface. The transplantation of *B2M^−/−^* cyiPSCs in immunodeficient mice resulted in teratoma that contained cartilage, indicating that the lack of MHC class I molecules on the cell surface affects neither the pluripotency nor the chondrogenic differentiation capacity of cyiPSCs. By modifying the chondrogenic differentiation protocol for human iPSCs, we succeeded at differentiating *B2M^+/+^* and *B2M^−/−^* cyiPSCs toward chondrocytes followed by cartilage formation *in vitro*, as indicated by histological analysis showing that *B2M^+/+^* and *B2M^−/−^* cyiPSC-derived cartilage were positively stained with safranin O and expressed type II collagen. Flow cytometry analysis confirmed that MHC class I molecules were not expressed on the cell surface of *B2M^−/−^* chondrocytes isolated from *B2M^−/−^* cyiPSC-derived cartilage. An *in vitro* mixed lymphocyte reaction assay showed that neither *B2M^+/+^* nor *B2M^−/−^* cyiPSC-derived cartilage cells stimulated the proliferation of allogeneic peripheral blood mononuclear cells. On the contrary, osteochondral defects in monkey knee joints that received allogeneic transplantations of cyiPSC-derived cartilage showed an accumulation of leukocytes with more natural killer cells around *B2M^−/−^* cyiPSC-derived cartilage than *B2M^+/+^* cartilage, suggesting complex mechanisms in the immune reaction of allogeneic cartilage transplanted in osteochondral defects *in vivo*.

**Impact statement:**

The transplantation of allogeneic induced pluripotent stem cell (iPSC)-derived cartilage is expected to treat articular cartilage damage, although the effects of major histocompatibility complex (MHC) in immunological reactions have not been well studied. We succeeded at creating *B2M^−/−^* cynomolgus monkey (cy)iPSCs and cyiPSC-derived cartilage that lack MHC class I molecules on the cell surface. *B2M^−/−^* cyiPSC-derived cartilage cells did not stimulate the proliferation of allogeneic peripheral blood mononuclear cells *in vitro*. On the contrary, the transplantation of *B2M^−/−^* cyiPSC-derived cartilage into osteochondral defects in monkey knee joints resulted in survival of transplants and accumulation of leukocytes, including natural killer cells, suggesting complex mechanisms for the immune reaction.

## Introduction

Articular cartilage resides at the end of bone, where it acts as a lubricant to ensure smooth joint motion. Cartilage has limited regenerative capacity, and its damage tends to result in a degenerative condition, impairing joint function. Cell therapies, including the implantation of chondrocytes or mesenchymal stem/stromal cells (MSCs), have been performed against the focal damage of articular cartilage, but, after expansion in culture, chondrocytes lose their chondrocytic character and MSCs lose their differentiation potential, compromising the formation of quality cartilage. In addition, expansion endows the senescent phenotype, which may affect the metabolism of these cells.

Cartilage is considered immunoprivileged,^1,2^ because it is avascular and because chondrocytes are surrounded by the extracellular matrix (ECM). The ECM blocks immune cells from contacting chondrocytes, thus avoiding immunological reactions even in allogeneic conditions. Accordingly, allogeneic cartilage harvested from juveniles^2–6^ have been transplanted into patients with articular cartilage damage without matching human leukocyte antigen (HLA) types or the use of immunosuppressive agents. However, donors are rare, and their allogeneic cartilage are heterogeneous in quality and risk the transmission of disease.

Induced pluripotent stem cells (iPSCs)^7,8^ are a potential alternative source for allogeneic transplantation. Because of their pluripotency and self-renewal, human iPSCs can be differentiated into abundant chondrocytes that secrete ECM and thus form scaffoldless cartilage tissue.^9–11^ Theoretically, iPSC-derived cartilage with uniform quality can be provided at almost infinite amounts due to the self-renew capacity of iPSCs, which overcomes the problems associated with current treatments for cartilage damage. Chondrocytes are segregated by the ECM in iPSC-derived cartilage, just like in native cartilage. Accordingly, human iPSC-derived cartilage does not stimulate the proliferation of allogeneic lymphocytes in mixed lymphocyte reaction assays.^12^ These findings suggest that the transplantation of allogeneic iPSC-derived cartilage could have medical benefit.

However, although both cartilage and iPSC-derived cartilage appear to have low immunogenicity, chondrocytes express major histocompatibility complex (MHC; HLA in humans) class I when treated with interferon γ (IFNγ).^2,12^ In contrast, MHC class II are not expressed in chondrocytes regardless of IFNγ treatment.^2,12^ Importantly, MHC class I risk inducing immune reactions. Therefore, when the cartilage is degraded and the chondrocytes are exposed, which is typical in arthritic conditions, immune reactions can occur. Furthermore, allogeneic cartilage transplantation into osteochondral defects, where the transplanted cartilage is exposed to abundant blood flow in the bone marrow, can further increase the risk of an immune reaction. To assess this risk, the effects of MHC class I on the cell surface in the transplantation of allogeneic cartilage or chondrocytes need to be investigated scientifically.

To evade immune reactions associated with the transplantation of allogeneic iPSC-derived cells, iPSCs with reduced immunogenicity, such as those made from HLA-homozygous individuals or genetically modified for HLA depletion, are under development.^13^ In addition, TALENs,^14^ CRISPR/Cas9,^15–17^ and vector-mediated gene targeting technology^18^ have been used to inactivate the *B2M* gene to deplete HLA-I. The simultaneous knockout of HLA-A/B/C has also been reported.^19^ Because natural killer (NK) cells reject HLA-depleted iPSC-derived allografts, the knock-in of HLA-E was done to prevent HLA-depleted ESCs from eliciting an immune response from either CD8^+^ T cells or NK cells.^18^ Finally, the targeted disruption of HLA-A and -B alleles in iPSCs reduces the lysis caused by T cells and NK cells.^17^

Before moving to the application of HLA-depleted iPSCs in clinical settings to treat articular cartilage damage, the effects of these HLA-depleted iPSC-derived chondrocytes should be examined in appropriate animal models in pre-clinical tests. Monkeys have a similar MHC structure as HLAs, thus making a good model. Therefore, in the present study, we generated cynomolgus monkey (crab-eating monkey [Macaca fascicularis]) iPSC (cyiPSC)-derived cartilage lacking MHC class I molecules on the cell surface and studied their chondrogenic and immunogenic properties.

## Materials and Methods

### Ethics statement

All methods were carried out in accordance with relevant guidelines and regulations. Experiments using recombinant DNA were approved by the Recombinant DNA Experiments Safety Committee of Kyoto University. All animal experiments were approved by the Institutional Animal Committee of Kyoto University.

### Monkey iPSCs and genome editing at the *B2M* locus

The wild-type cyiPSC line 1123C1 was previously described.^20^ cyiPSCs were maintained in AK-02N medium (Ajinomoto).

**FIG. 1.**
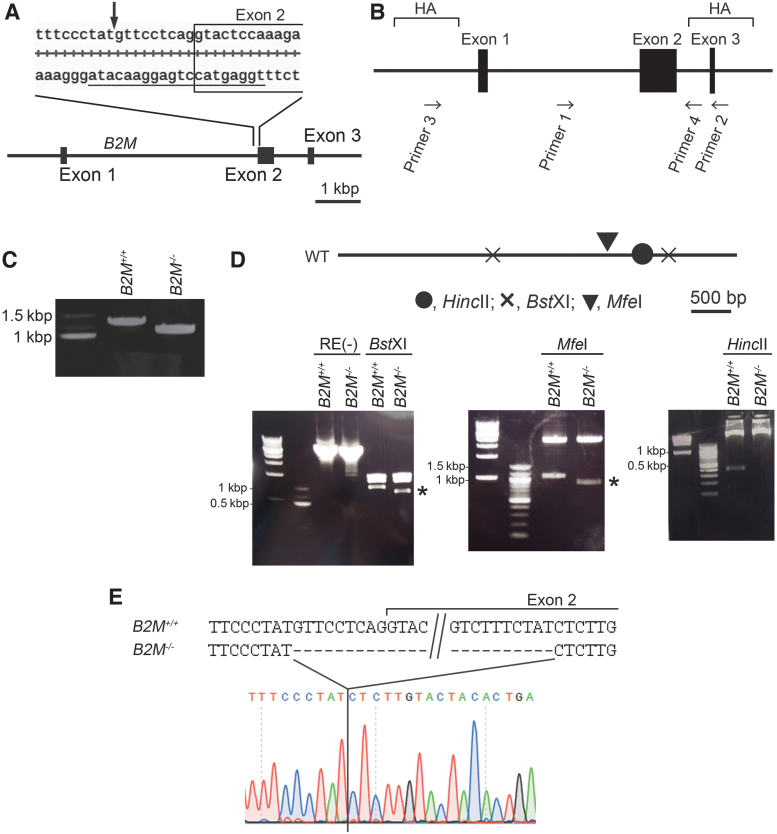
Inactivation of *B2M* gene in cyiPSCs using the CRISPR/Cas9 system. **(A)** Schematic representation of the target sequence recognized by the guide RNA. The target sequence for the guide RNA is underlined. The *arrow* indicates the site of the double strand break. **(B)** Locations of the sequences corresponding to the primers used for the PCR amplification. **(C)** PCR amplification with primers 1 and 2 using genomic DNA extracted from *B2M^+/+^* or *B2M^−/−^* cyiPSCs. The PCR products were separated on an agarose gel. The expected size of the PCR product from *B2M^+/+^* genomic DNA is 1205 bp. **(D)** PCR amplification with primers 3 and 4 using genomic DNA extracted from *B2M^+/+^* or *B2M^-/−^* iPSCs. The PCR products were digested with restriction enzymes. *Top*, Schematic representation of the expected PCR products with primers 3 and 4 from the wild-type allele and locations of the sites recognized by the restriction enzymes *Bst*XI, *Mfe*I, and *Hin*cII. *Bottom*, PCR products digested with the restriction enzymes were separated on an agarose gel during electrophoresis. *RE (−)*, without digestion. *Asterisks* indicate fragments of different size between *B2M^+/+^* and *B2M^−/−^* cyiPSCs. **(E)** The PCR product from *B2M^−/−^* genomic DNA was subjected to direct sequencing. B2M, β2 microglobulin; iPSCs, induced pluripotent stem cells; polymerase chain reaction (PCR).

CRISPR-Cas9 constructs were designed based on a previous report.^21^ To inactivate *B2M*, we aimed at disrupting exon 2 of the cynomolgus monkey *B2M* gene. The guide RNA sequence was UAUGUUCCUCAGGUACUCCA, which corresponds to the sequence at the 5′-end of exon 2 (Fig. 1A). The guide oligo DNAs (Table 1) corresponding to the guide RNA sequence were annealed and ligated to pSpCas9(BB)-2A-Puro.^21^ *BbsI* was then used to digest pSpCas9(BB)-2A-Puro. DNA vectors (3.3 μg) encoding the guide RNA for *B2M* and Cas9 were introduced into 1.0 × 10^6^ cyiPSCs in 100 μL OPTI-MEM by a nucleofection system following the manufacturer's instructions (Nepa21, Nepa Gene). Electric pulses (175 V, 5 ms) were used. Two days after the nucleofection, the cells were cultured with puromycin. Eight days after the nucleofection, 39 colonies were picked up, and the cells in each colony were expanded. Genomic DNAs were extracted from the cells and subjected to polymerase chain reaction (PCR) analysis.

**Table 1.** List of Primers

Name	Sequence
Guide oligo DNAs used for genome editing	
sgRNA	
Top	CACCGTGGAGTACCTGAGGAACATA
Bottom	AAACTATGTTCCTCAGGTACTCCAC
Genomic PCR	
Primer1	GGGGTGGAAACAGAGTACAGTAACATGAGTAATTTGATGG
Primer2	CTCAGATACCAATCCAGCCAGAGAGTACTGG
Primer3	TAGCAAGTCACTTAGTATCTCTGGGACCAGTTTGC
Primer4	GCAATCTCTCAGCAGATGCCACTAATCTGATCT
EGFP	
FW	TTACTTGTACAGCTCGTCCATGCCGAGAGT
RV	ATGGTGAGCAAGGGCGAGGAGC
RT-qPCR	
*POU5F1*	
FW	GAGAACAATGAGAACCTTCAGGAGA
RV	TTCTGGCGCCGGTTACAGAACCA
*NANOG*	
FW	CAGAAGGCCTCAGCACCT
RV	GACTGTTCCAGGCCTGATTGTT
FW	AGGCTATCCAGCGTACTCCA
*B2M*	
RV	CGGCAGGCATACTCATCTTT
*ACTB*	
FW	TGAAGATCCTCACTGAGCGC
RV	CTCTTCTCCAGGGAGGAGCT
*GAPDH*	
FW	AATCCCATCACCATCTTCCAGGAG
RV	CACCCTGTTGCTGTAGCCAAATTC
*SOX9*	
FW	GATGGCCGAGATGATCCTAA
RV	GTTGATTTCGCTGCTCCATT
*COL2A1*	
FW	ACCTTCCTACGCCTGCTTTC
RV	GTCCAGGTAGGCAATGCTGT
*ACAN*	
FW	TTCTGCTTCCGAGGGGTTTC
RV	TAGCGATCCAGTCCTCCACA
*SOX2*	
FW	ATATGAACGGCTGGAGCAAC
RV	ATGTAGGTCTGCGAGCTGGT

FW, forward; RV, reverse.

RT-qPCR, reverse transcription quantitative PCR.

### Chondrogenic differentiation of cyiPSCs followed by cartilage formation

The cyiPSCs were chondrogenically differentiated to produce cartilage using a previously described method for human iPSCs^9^ with one minor modification. In brief, after chondrogenic differentiation, the cells were transferred into suspension culture to induce ECM secretion and form cartilaginous particles 1–3 mm in diameter. Cells and particles were cultured in chondrogenic medium [Dulbecco's modified Eagle's medium (DMEM) (Sigma, St. Louis) with 1% ITS-X (Thermo Fisher Scientific, Waltham), 1% fetal bovine serum (FBS) (Thermo Fisher Scientific), 1 × 10^−4^ M nonessential amino acids (Thermo Fisher Scientific), 1 mM Na pyruvate (Thermo Fisher Scientific), 50 U penicillin, 50 mg/mL streptomycin (1% PC/SM, Thermo Fisher Scientific), 0.25 μg/mL amphotericin B (Thermo Fisher Scientific), 50 μg/mL ascorbic acid (Nacalai), 1 μM rosuvastatin (BioVision, Milpitas), 10 ng/mL BMP2 (Peprotech), 10 ng/mL transforming growth factor beta 1 (TGFβ1) (Peprotech), and 10 ng/mL GDF5 (BioVision)]. For human iPSCs, the chondrogenic medium is typically replaced with the conventional medium (DMEM supplemented with 10% FBS and 1% PC/SM) at 6 weeks after the chondrogenic differentiation.^9^ In contrast, cyiPSCs were kept in chondrogenic medium after the chondrogenic differentiation until the analysis.

### Isolation of cells from cyiPSC-derived cartilage

cyiPSC-derived cartilage was treated with 0.25% trypsin-EDTA (Thermo Fisher Scientific) for 1 h and subsequently with 4 mg/mL collagenase D (Roch) in DMEM supplemented with 1% PC/SM for 3 h. After washing, the cells were suspended in DMEM supplemented with 1% ITS-X, 10% FBS, 1 × 10^−4^ M nonessential amino acids, 1 mM Na pyruvate, 1% PC/SM, 0.25 μg/mL amphotericin B, 50 μg/mL ascorbic acid, 10 ng/mL TGFβ1, and 100 nM dexamethasone. Afterward, the cells were analyzed by flow cytometry.

### Cynomolgus monkey kidney epithelial-like cells (MK.P3)

The cynomolgus monkey kidney cell line MK.P3 (registry No. JCRB0607.1) was obtained from JCRB Cell Bank (Osaka, Japan)^22^ and used as a nonpluripotent control. Cryo-preserved MK.P3 was thawed and plated in DMEM-F12 (Sigma) supplemented with 10% FBS and 1% PC/SM.

### PCR digestion with restriction enzymes and sequencing

We obtained and analyzed genomic DNA from cyiPSCs using the DNeasy^®^ Blood & Tissue Kit (Qiagen, Hilden) and PCR, respectively. PCR was performed using KOD plus Neo (Toyobo). Electrophoresis was performed with 100 bp and 1 kbp DNA ladders (New England Biolabs, Ipswich). The PCR products were purified using NucleoSpin^®^ Gel and PCR Clean-up (Macherey-Nagel) and digested by three restriction enzymes: *Hin*cII, *Bts*XI and *Mfe*I (New England Biolabs). Table 1 lists the primers used.

For direct sequencing of the PCR products, we obtained DNA fragments from an agarose gel using NucleoSpin Gel and PCR Clean-up. The fragments were analyzed using a Big Dye Terminator Kit (Thermo Fisher Scientific) and Applied Biosystems^®^ 3500 × L (Thermo Fisher Scientific). The sequence data were analyzed using SnapGene^®^ ver 4.0.8 (GSL Biotech LLC, Chicago).

### mRNA expression

cyiPSC-derived cartilage were frozen in liquid nitrogen and crushed by a Multi-Beads Shocker (Yasui Kikai, Japan). Total RNAs were extracted using RNeasy (Qiagen). For quantitative reverse transcription PCR (RT-PCR), 200 ng total RNA was reverse transcribed into first-strand cDNA using ReverTra Ace (Toyobo) and an oligo(dT)20 primer. The PCR amplification was performed using a KAPA SYBR FAST qPCR Master Mix ABI prism Kit (KAPA Biosystems, Wilmington) and StepOnePlus Real-Time PCR System (Thermo Fisher Scientific). Table 1 lists the PCR primers used.^23,24^ The RNA expression levels were normalized to the level of *GAPDH* or *ACTB* expression, and the results indicate the relative expression of the molecules.

### Western Blotting

We obtained proteins from the cells using lysis buffer and performed western blotting using Nupage Novex Bis Tris Gel (Thermo Fisher Scientific). Table 2 lists the antibodies used. The signals were recorded using ImageQuant LAS 4000 (GE Healthcare).

**Table 2.** List of Antibodies

Antibody	Species	Dilution	Catalog No.	Source
Flow cytometry analysis				
Anti-HLA class I-PE		1:100	Orb44579	Biorbyt
Anti-mouse IgG2a (isotype control)-PE		1:100	M076-5	MBL
Anti-human CD3-PE-Cy7		1:20	557749	BD Biosciences
Anti-human CD4-APC		1:20	317416	BioLegend
Anti-human CD8-PE		1:5	555367	BD Biosciences
Anti-CD159a (NKG2a)-APC		1:10	A60797	BECKMAN COULTER
Immunocytochemical analysis				
Anti-Nanog (D73G4)	Rabbit	1:400	#4903	Cell Signaling
Anti-Oct3/4	Mouse	1:250	Sc-5279	Santa cruz
Anti-GFP	Rat	1:500	04404-26	Nacalai
Alexa Fluor 546 goat anti-rabbit IgG(H+L)	Goat	1:500	A11010	Life Tech
Alexa Fluor 488 goat anti-rat IgG(H+L)	Goat	1:500	A11006	Life Tech
Alexa Fluor 546 goat anti-mouse IgG(H+L)	Goat	1:500	A11003	Life Tech
Immunohistochemical Analysis				
Anti-Collagen II	Mouse	1:1000	MS235P0	Thermo
Anti-CD3	Rat	1:100	Ab11089	abcam
Anti-NKG2A	Rabbit	1:2000	Ab260035	abcam
Anti-mouse immunoglobulin HRP			K1497	DAKO
Alexa Fluor 488 goat anti-mouse IgG(H+L)	Goat	1:1000	A11029	Life Tech
Alexa Fluor 488 goat anti-rabbit IgG(H+L)	Goat	1:1000	A11008	Thermo
Alexa Fluor 488 goat anti-rat IgG(H+L)	Goat	1:1000	A11006	Thermo
Western Blotting				
Beta-2-microglobulin (D8P1H) Rabbit mAb	Rabbit	1:2000	12851S	Cell signaling
Anti-MHC class I+HLA A+HLA B	mouse	1:1000	Ab134189	abcam
Anti Gapdh	mouse	1:1000	Sc-47724	Santa Cruz
Goat anti-mouse IgG-HRP	Goat	1:2000	Sc-2005	Santa Cruz
Goat anti-rabbit IgG-HRP	Goat	1:2000	Sc-2004	Santa Cruz

HLA, human leukocyte antigen; IgG-HRP, immunoglobulin G-horseradish peroxidase; PE, Phosphatidylethanolamine; MHC, major histocompatibility complex.

### Immunocytochemical analysis

cyiPSCs were cultured on a culture dish and fixed using 4% paraformaldehyde (Nacalai). After washing with phosphate-buffered saline (PBS), the cells were incubated at 4°C with primary antibodies overnight. After washing again with PBS, the cells were incubated at room temperature for 1 h and analyzed microscopically (Eclipse Ti, Nikon).

### Flow cytometry analysis

The expression of cell surface markers was analyzed by flow cytometry using a FACS Aria II flow cytometer (BD Bioscience, California) and FlowJo version 10 software (FlowJo). cyiPSCs and cells isolated from cyiPSC-derived cartilage were stained with anti-human antibodies at 4°C for 30 min. Table 2 lists the antibodies used. The MHC expression was also analyzed after treating the cells with 25 ng/mL IFNγ (Sigma) for 72 h. Isotype control antibodies were used (Table 2).

### Histological and immunohistochemical analysis

Samples were fixed with 4% paraformaldehyde, processed and embedded in paraffin. Semiserial sections were prepared for staining with hematoxylin and eosin or Safranin O-fast green-iron hematoxylin (Safranin O) or immunostaining with specific antibodies. Anti-type II collagen antibodies were detected using a CSA II Biotin-free Tyramide Signal Amplification System Kit (Agilent Technologies, California) and DAB as the chromogen. For the anti-CD3 antibodies and anti-NKG2A antibodies, immune complexes were detected using secondary antibodies conjugated to Alexa Fluor 488. Antigens were unmasked by treatment with hyaluronidase and EDTA. Table 2 lists the antibodies used.

### Teratoma formation

To form teratomas, cyiPSCs were injected into the testicular capsules of 4-week-old C.B-17/Icr-scid/scid Jcl male mice. Twelve weeks later, the tumors were recovered and subjected to histological analysis.

### Mixed lymphocyte assay

5 × 10^5^ cynomolgus monkey peripheral blood mononuclear cells (PBMCs; iQ Biosciences) were cocultured with 1 × 10^5^ cells isolated from cyiPSC-derived cartilage in RPMI1640 supplemented with 10% FBS in 1 well of a 96-well plate for 110 h in the presence or absence of 25 ng/mL IFNγ. Then, the proliferation of PBMCs was analyzed using a CellTrace™ CFSE Cell Proliferation Kit (Thermo Fisher Scientific). In brief, PBMCs were pretreated with carboxyfluorescein diacetate succinimidyl ester (CFSE) before the start of the coculture. The number of PBMC divisions was analyzed flow cytometry after the coculture. As for a positive control, PBMCs were cultured in the presence of 1 μg/mL anti-CD3 antibody (Thermo Fisher Scientific) for 110 h.

Next, 3 × 10^5^ PBMCs were cocultured with 3 × 10^4^ cyiPSCs in RPMI1640 supplemented with 10% FBS and 25 ng/mL IFNγ in 1 well of a 96-well plate for 130 h. The proliferation of CD3^+^ CD4^+^ T cells, CD3^+^ CD8^+^ T cells, and CD3^−^ CD159a^+^ NK cells was analyzed by CFSE labeling.

### Transplantation of cyiPSC-derived cartilage into osteochondral defects in cynomolgus monkey

Cynomolgus monkeys (3–4 years old) were purchased from Ina Research (Nagano, Japan). We created osteochondral defects (1.5 mm in diameter and 1–2 mm in depth) in the trochlea of the distal femur in the right knee joints of four male cynomolgus monkeys. We transplanted *B2M^+/+^* cyiPSC-derived cartilage in the defects in two monkeys and *B2M^−/−^* cyiPSC-derived cartilage in the defects in the other two monkeys. After surgery, antibiotics and buprenorphine hydrochloride (0.1 mg/body) were intramuscularly injected for 3 days. Monkeys were sacrificed 4 weeks later by injecting pentobarbital sodium (100 mg/kg) under deep anesthesia, and transplanted sites were harvested and analyzed histologically. Samples were decalcified with KCX (FALMA).

### Statistical analysis

The data are shown as averages and standard deviations. In this study, we used two-tailed Student's *t*-tests. *p*-Values <0.05 were considered statistically significant.

## Results

### *B2M* gene deletion in cyiPSCs

To be expressed on the cell surface, MHC class I molecules form multimers with β2 microglobulin (B2M).^25^ Therefore, we deleted the B2M gene (*B2M*) using the CRISPR-Cas9 system (Fig. 1A). A previous report on mice showed that deletion of exon 2 of *B2M* prevents the expression of B2M protein.^26^ Therefore, the target sequence for the guide RNA was designed at around the 5′ end of exon 2 (Fig. 1A). After the nucleofection of 1123C1 cyiPSCs with the guide RNA and Cas9, we obtained 39 clone lines.

We confirmed that the clone 1123C1-te lacked the *B2M* gene (*B2M^−/−^* cyiPSCs). We extracted genomic DNA and subjected them to PCR using primers 1 and 2 (Fig. 1B). *B2M^−/−^* cyiPSCs showed a unique amplification product whose size was smaller than the product generated from wild-type (*B2M^+/+^*) cyiPSCs by around 200 bp (Fig. 1C).

Genomic PCR analysis using primers 3 and 4 (Fig. 1B) revealed that the size of the PCR product generated from *B2M^−/−^* cyiPSCs was identical or slightly shorter than that from *B2M^+/+^* cyiPSCs [Fig. 1D, RE(−)]. We then digested the PCR product with restriction enzymes. Digestion with *Bst*XI showed a restriction pattern similar with that of *B2M^+/+^* cyiPSCs except for one fragment (Fig. 1D). Digestion with *Mfe*I also resulted in a restriction pattern similar with *B2M^+/+^* cyiPSCs except for one fragment (Fig. 1D). In both the *Bst*XI and *Mfe*I digestions, the sizes of the one exceptional fragment (Fig. 1D, asterisks) was around 200 bp shorter than the fragment from *B2M^+/+^* cyiPSCs. Digestion of the PCR product from *B2M^+/+^* cyiPSCs with *Hin*cII produced expected fragments of 4696 and 374 bp, but that from *B2M^−/−^* cyiPSCs produced a single fragment of around 5 kbp (Fig. 1D). These results from the restriction enzyme analysis suggest that *B2M* lost an ∼200-bp fragment containing the *Hin*cII site (Fig. 1D) and locates between the sequences corresponding to primers 1 and 2 (Fig. 1B). Direct sequencing analysis of the PCR products using primers 1 and 2 revealed that the product from *B2M^−/−^* cyiPSCs lacked a 191 bp stretch (Fig. 1E) that includes the target sequence (Fig. 1A) of the guide RNA. The splice acceptor site of exon 2 and the 5′ part of the 182 bp stretch of exon 2 were deleted from *B2M* in *B2M^−/−^* (Fig. 1E). A single product from the PCR amplification and unique nucleotide signals in the direct sequencing analysis suggested that the identical 191 bp stretch was deleted from both alleles in *B2M^−/−^* cyiPSCs or that one allele lacks the large fragment. The deletion should cause either a frameshift mutation in exon 2 or exon 2 skipping due to the loss of the acceptor site for splicing.

### Expression analysis of MHC class I molecules in *B2M^−/−^* cyiPSCs

**FIG. 2.**
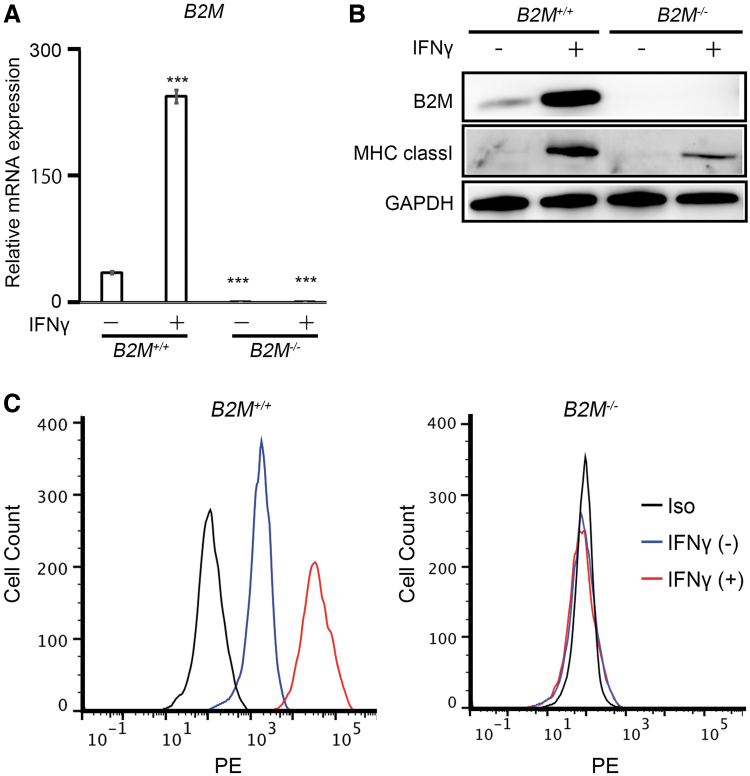
Expression analysis of B2M and MHC class I molecules in *B2M^+/+^* and *B2M^−/−^* cyiPSCs. **(A)** RT-PCR expression analysis of *B2M* mRNA. The RNA expression levels were normalized to the level of *ACTB* expression. Error bars denote means ± SD (*n* = 3 technical replicates). ***p < 0.001 by the two-tailed Student's *t*-tests compared with *B2M^+/+^* cyiPSCs without IFNγ treatment. Data are representative of three independent experiments. **(B)** Western blot analysis of MHC class I molecules with or without IFNγ treatment. The data are representative of two independent experiments. **(C)** Flow cytometry analysis of cells without (*blue line*) or with (*red line*) IFNγ stimulation using anti-MHC class I antibodies. *Black lines* represent data from the isotype control. Data are representative of two independent experiments. IFNγ, interferon γ; MHC, major histocompatibility complex; reverse transcription PCR (RT-PCR); SD, standard deviation.

RT-PCR analysis using the forward primer, which corresponds to the junction of exon 1 and exon 2, indicated that amplification did not occur in *B2M^−/−^* cyiPSCs regardless of IFNγ stimulation (Fig. 2A). Western blot analysis confirmed that *B2M^−/−^* cyiPSCs did not express B2M (Fig. 2B). In addition, the expression level of B2M protein was elevated by IFNγ stimulation in *B2M^+/+^* cyiPSCs, but not in *B2M^−/−^* cyiPSCs. IFNγ induced the expression of MHC class 1 proteins in *B2M^+/+^* cyiPSCs and to a lesser extent in *B2M^−/−^* cyiPSCs. Flow cytometry analysis indicated that *B2M^+/+^* cyiPSCs expressed MHC class I molecules on the cell surface (Fig. 2C, left, blue line) and that IFNγ increased the expression (Fig. 2C, left, red line). On the contrary, no *B2M^−/−^* cyiPSCs expressed MHC class I molecules on the cell surface regardless of IFNγ stimulation (Fig. 2C, right); thus, MHC class I protein indicated in the western blot (Fig. 2B) was present intracellularly. These results are consistent with the observed absence of B2M, which is needed for MHC class I proteins to form a complex and be expressed on the cell surface.

### Pluripotency of *B2M^−/−^* cyiPSCs

**FIG. 3.**
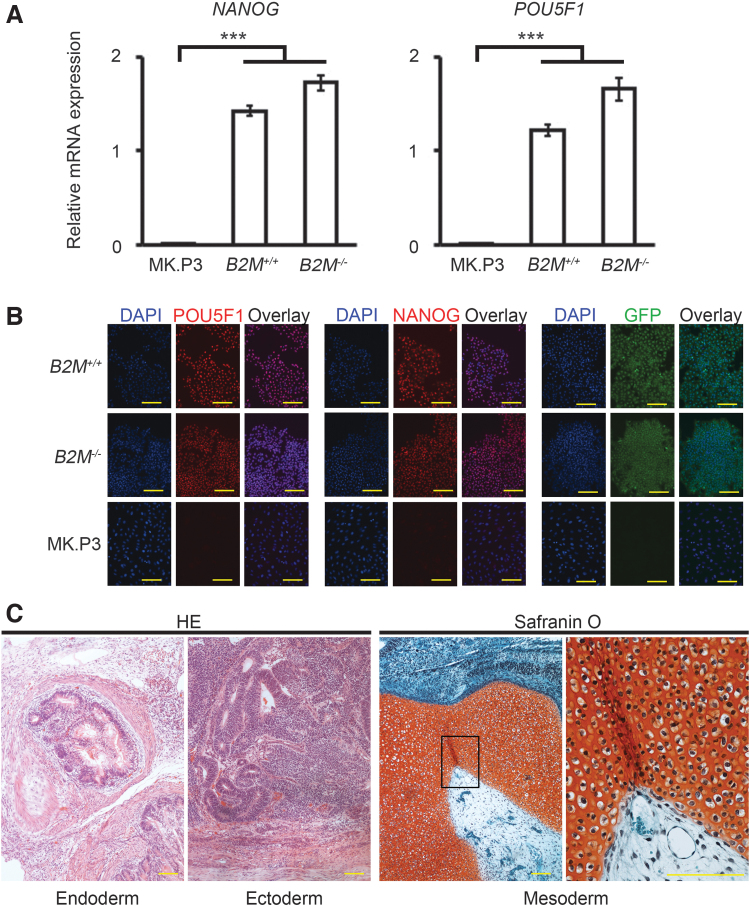
Pluripotency of *B2M^−/−^* cyiPSCs. **(A)** RT-PCR expression analysis of *POU5F1* and *NANOG* mRNAs. The RNA expression levels were normalized to the level of *GAPDH* expression. Error bars denote means ± SD (*n* = 3 experiments). ***p < 0.001, comparison with MK.P3 (cynomolgus monkey kidney epithelial-like cells, negative control; two-tailed Student's *t*-test). **(B)** Immunocytochemical analysis of POU5F1, NANOG, and GFP in *B2M^+/+^* cyiPSCs, *B2M^−/−^* cyiPSCs, and MK.P3. The data are representative of four independent experiments. **(C)** *B2M^−/−^* cyiPSCs were inoculated on the testicular capsules of 6 immunodeficient mice. The mice were sacrificed 3 months later, and samples were subjected to histological analysis. Tumors were found in all six mice. Four tumors contained tissues of all three germ layers. Hematoxylin-eosin and safranin O-fast *green*-iron hematoxylin staining. Magnification of the *boxed region* in the *third panel* is shown in the *right panel*. Scale bars, 100 μm. B2M, β2 microglobulin.

RT-PCR and immunocytochemistry analyses indicated that the expression levels of pluripotent markers, such as POU5F1 and NANOG, in *B2M^−/−^* cyiPSCs were not significantly different from those in *B2M^+/+^* cyiPSCs (Fig. 3A, B). *B2M^−/−^* cyiPSCs formed teratoma containing tissues of all three germ layers 3 months after inoculation into the testicular capsules of immunodeficient mice (Fig. 3C), indicating that *B2M^−/−^* cyiPSCs have pluripotency.

### Differentiation of *B2M^−/−^* into cartilage

**FIG. 4.**
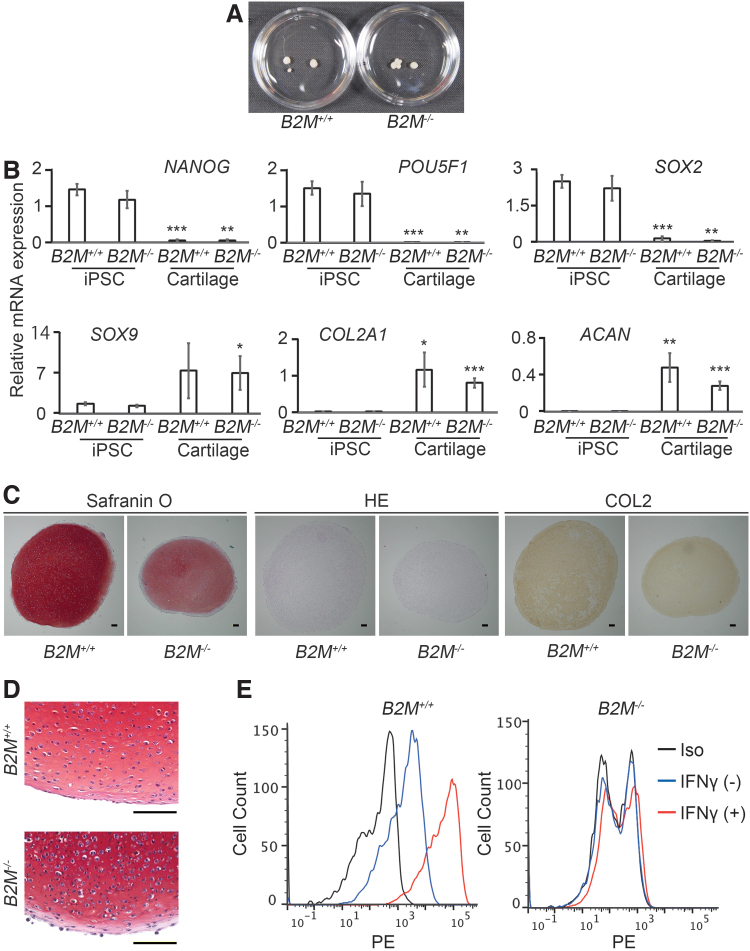
Expression analysis of pluripotent markers, chondrocyte markers, and MHC class I molecules in *B2M^+/+^* and *B2M^−/−^* cyiPSC-derived cartilage. **(A)** Particles created from *B2M^+/+^* and *B2M^−/−^* cyiPSCs by chondrogenic differentiation. The diameter of the culture dish is 3.5 cm. **(B)** RT-PCR expression analysis of pluripotent and chondrocyte markers in *B2M^+/+^* and *B2M^−/−^* cyiPSCs and cartilage derived from them. The RNA expression levels were normalized to the level of *GAPDH* expression. Error bars denote means ± SD (*n* = 3 culture dishes). *p < 0.05, **p < 0.01, and ***p < 0.001 by two-tailed Student's *t*-tests. Comparisons were made between iPSCs and cartilage from the same line. **(C)** Semiserial sections of cyiPSC-derived cartilage were stained with safranin O-Fast *green*-iron hematoxylin and hematoxylin-eosin and immunostained with anti-type II collagen antibody. The data are representative of seven cyiPSC-derived cartilage. **(D)** Magnified images of sections of cyiPSC-derived cartilage stained with safranin O-Fast *green*-iron hematoxylin. **(E)** cyiPSC-derived cartilage were digested with trypsin-EDTA and collagenase D, and the released cells were subjected to flow cytometry analysis. *Red* and *blue lines* indicate with or without IFNγ stimulation, respectively, using anti-MHC class I antibodies. *Black lines* represent data from the isotype control. Data are representative of two independent experiments. The digestion procedure might have damaged the cells, thus generating the double peaks. Scale bars, 100 μm.

Chondrogenic differentiation of *B2M^+/+^* or *B2M^−/−^* cyiPSCs resulted in the formation of spherical particles 1–3 mm in diameter (Fig. 4A). RT-PCR expression analysis revealed the expression levels of genes associated with chondrogenesis, including *COL2A1* and *ACAN*, were elevated, and the expression levels of genes associated with pluripotency were drastically decreased in the particles compared with cyiPSCs (Fig. 4B). Histological analysis revealed that the particles consisted of cells and ECM based on positive staining with safranin O and immunostaining with anti-type II collagen antibody (Fig. 4C, D). These results collectively suggest that both *B2M^+/+^* and *B2M^−/−^* cyiPSCs can be chondrogenically differentiated to form cartilage.

### Expression analysis of MHC class I molecules in *B2M^−/−^* cyiPSC-derived cartilage

As with cyiPSCs, flow cytometry analysis indicated that cells isolated from *B2M^+/+^* cyiPSC-derived cartilage expressed MHC class I molecules weakly on the cell surface (Fig. 4E, left, blue line) and that treatment with IFNγ increased the expression level (Fig. 4E, left, red line). On the contrary, no cells isolated from *B2M^−/−^* cyiPSC-derived cartilage expressed MHC class I molecules on the cell surface regardless of IFNγ stimulation (Fig. 4E, right).

### *In vitro* mixed lymphocyte reaction assay

**FIG. 5.**
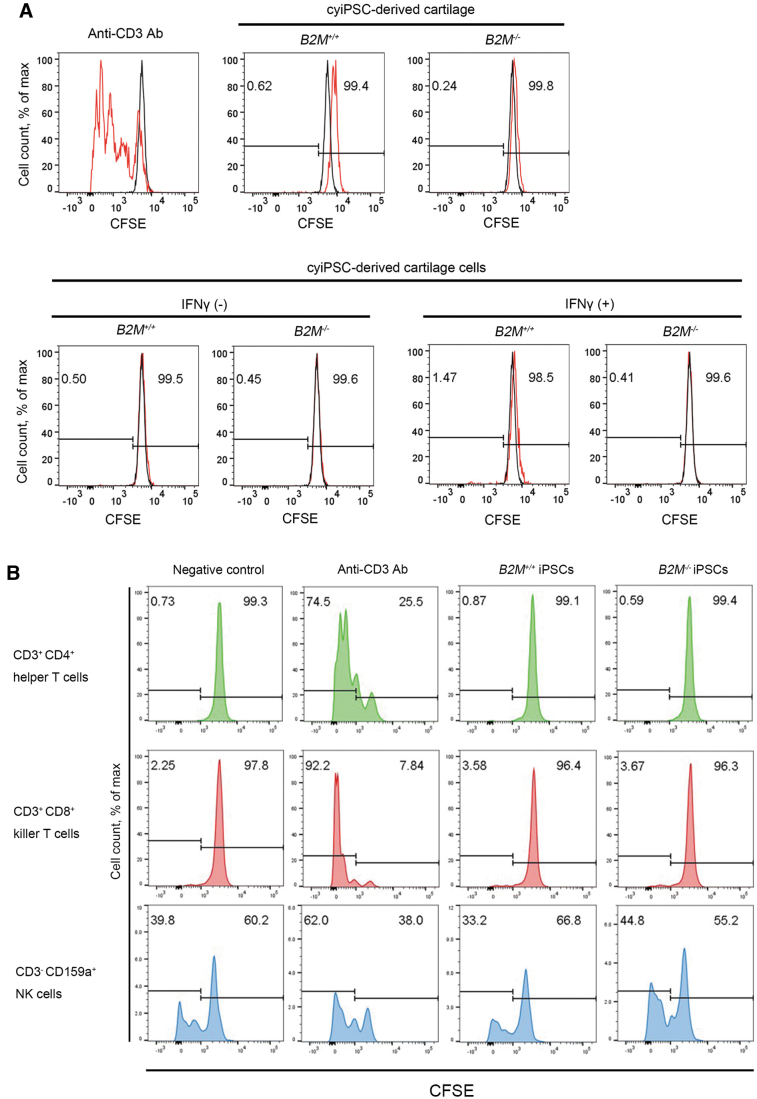
*In vitro* mixed lymphocyte reaction assay using *B2M^−/−^* cyiPSC-derived cartilage and *B2M^−/−^* cyiPSC-derived cartilage cells. **(A)** PBMCs were pretreated with CFSE. *Top*, *left*, allogeneic PBMCs were cultured with anti-CD3 antibody, which served as a positive control. *Top*, *middle*, *and right*, allogeneic PBMCs were cocultured with *B2M^+/+^* or *B2M^−/−^* cyiPSC-derived cartilage. *Bottom*, *B2M^+/+^* or *B2M^−/−^* cyiPSC-derived cartilage were digested with trypsin-EDTA and collagenase D, and the released cells were cocultured with allogeneic PBMCs in the absence or presence of IFNγ. **(B)** *B2M^+/+^* or *B2M^−/−^* cyiPSCs were cocultured with allogeneic PBMCs. The proliferation of CD3^+^ CD4^+^ T cells, CD3^+^ CD8^+^ T cells, and CD3^−^ CD159a^+^ NK cells was analyzed by CFSE labeling. PBMCs were cultured with anti-CD3 antibody as a positive control (*second column* from *left*). CFSE, carboxyfluorescein diacetate succinimidyl ester; PBMCs, peripheral blood mononuclear cells; NK, natural killer.

Next, we performed mixed lymphocyte reaction assays by coculturing allogeneic PBMCs with *B2M^+/+^* or *B2M^−/−^* cyiPSC-derived cartilage. An analysis using CFSE showed that the proliferation of PBMCs was stimulated by the addition of anti-CD3 antibody, which served as a positive control (Fig. 5A, top row, left panel). In coculture, neither *B2M^+/+^* nor *B2M^−/−^* cyiPSC-derived cartilage stimulated the proliferation of PBMCs (Fig. 5A, top row, middle, and right panel). The lack of proliferation may be because the ECM surrounds the chondrocytes in cyiPSC-derived cartilage, preventing the allogeneic PBMCs from directly contacting the chondrocytes.

Then we digested the ECM of *B2M^+/+^* and *B2M^−/−^* cyiPSC-derived cartilage and isolated the cells. The cells were subsequently subjected to coculture with allogeneic PBMCs. In this condition, *B2M^+/+^* and *B2M^−/−^* cyiPSC-derived cartilage cells directly contacted lymphocytes. However, neither *B2M^+/+^* nor *B2M^−/−^* cyiPSC-derived cartilage cells stimulated the proliferation of PBMCs regardless of IFNγ treatment (Fig. 5A, bottom row). These results suggest that the immunogenicity of both *B2M^+/+^* and *B2M^−/−^* cyiPSC-derived cartilage cells is below detectable levels in the *in vitro* mixed lymphocyte reaction assay.

In addition, mixed lymphocyte reaction assays using undifferentiated cyiPSCs indicated that CD3^+^ CD4^+^ helper T cells and CD3^+^ CD8^+^ killer T cells did not proliferate in the presence of either *B2M^+/+^* or *B2M^−/−^* cyiPSCs (Fig. 5B). On the contrary, CD3^−^ CD159a^+^ cells in the presence of *B2M^−/−^* cyiPSCs proliferated more than in the presence of *B2M^+/+^* cyiPSCs, suggesting that *B2M^−/−^* cyiPSCs stimulated NK cells (Fig. 5B).

### *In vivo* allogeneic transplantation of *B2M^−/−^* cyiPSC-derived cartilage in osteochondral defects of monkey knee joints

**FIG. 6.**
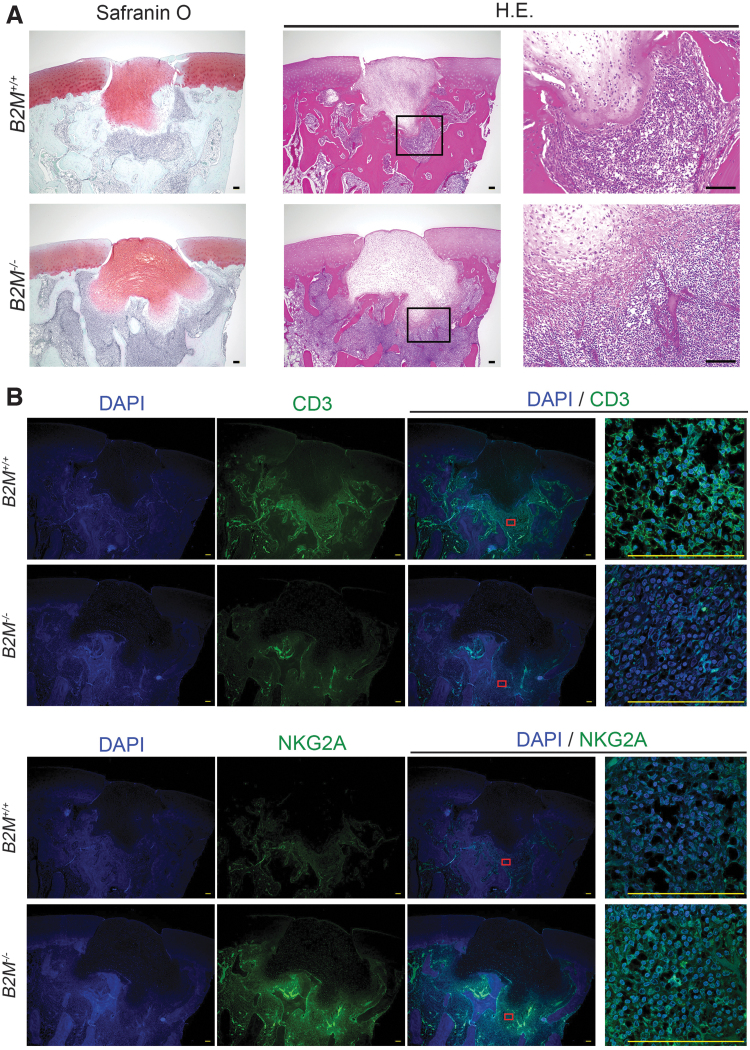
*In vivo* allogeneic transplantation of *B2M^−/−^* cyiPSC-derived cartilage. **(A)** Allogeneic *B2M^+/+^* cyiPSC-derived cartilage (*top row*) and *B2M^−/−^* cyiPSC-derived cartilage (*bottom row*) were transplanted into osteochondral defects created in the joint surface of the distal femur in the knee joints of monkeys. The transplanted sites were analyzed histologically 4 weeks after the transplantation. *Left*, Safranin O-fast *green*-iron hematoxylin staining. *Middle*, HE staining. *Right*, magnification of the *boxed regions* in the *middle panels*. **(B)** Immunohistochemical analysis of the semiserial sections in (A) using anti-CD3 antibody (*top two rows*) and anti NKG2A antibody (*bottom two rows*). Magnification of the *boxed regions* in the *third column* is shown in the *fourth column*. Scale bars, 100 μm. HE, hematoxylin and eosin.

Finally, we analyzed the *in vivo* immunogenicity of *B2M^−/−^* cyiPSC-derived cartilage by transplanting *B2M^+/+^* and *B2M^−/−^* cyiPSC-derived cartilage into osteochondral defects created in the joint surface of the distal femur in cynomolgus monkey knee joints. Four weeks after the transplantation, histological analysis revealed that both *B2M^+/+^* and *B2M^−/−^* cyiPSC-derived cartilage survived and were not rejected but that they also caused an accumulation of lymphocytes in the bone marrow (Fig. 6A). This preliminary study was performed in two monkeys for each *B2M^+/+^* and *B2M^−/−^* cartilage transplantation, and the lymphocyte accumulation was reproducible in all four monkeys, suggesting that the lack of MHC class I is not sufficient for eliminating immune reactions against iPSC-derived cartilage transplanted in osteochondral defects. To characterize the cell types that accumulated around transplanted cyiPSC-derived cartilage, we performed immunohistochemical analysis (Fig. 6B). Immunoreactivity against anti-CD3 antibody tended to be weaker around B2M^−/−^ cyiPSC-derived cartilage than around B2M^+/+^ cyiPSC-derived cartilage. On the contrary, immunoreactivity against anti-NKG2A antibody was stronger around B2M^−/−^ cyiPSC-derived cartilage than around B2M^+/+^ cyiPSC-derived cartilage, suggesting that NK cells rather than T cells accumulated around B2M^−/−^ cyiPSC-derived cartilage.

## Discussion

Allogeneic transplanted tissue elicits an immune response when recognized by the recipient's T lymphocytes as “non-self.”^27^ One mechanism involved in the rejection is the activation of lymphocytes by direct contact with intact MHC class I located on the cell surface of the transplanted allogeneic cells. In this study, we created a cyiPSC clone, in which *B2M* is deleted (*B2M^−/−^*). *B2M^−/−^* cyiPSCs expectedly lacked MHC class I expression on the cell surface, a property that did not affect the pluripotency of cyiPSCs. Indeed, we could generate cartilage efficiently from *B2M^−/−^* cyiPSCs as well as *B2M^+/+^* cyiPSCs. *B2M^−/−^* cyiPSC-derived cartilage cells lack MHC class I expression on their surface even in the presence of IFNγ. Considering the closeness in immune systems between human and monkey, the *B2M^−/−^* cyiPSCs- and *B2M^−/−^* cyiPSC-derived chondrocytes/cartilage created in this study should be useful for estimating the immunogenicity of transplants derived from allogeneic iPSCs and ways to control immune rejection in human.

The *in vitro* mixed lymphocyte reaction assay revealed that lymphocytes were not stimulated by chondrocytes isolated from *B2M^+/+^* cyiPSC-derived cartilage even in the presence of IFNγ. This result can be explained by chondrocytes suppressing the activation of T cells partly through the constitutive expression of B7 family members, which transmit inhibitory signals to T cells.^2^ Thus, *in vitro* mixed lymphocyte reaction assays may not be appropriate for demonstrating the immune evasion effects of *B2M^−/−^* cyiPSC-derived cartilage cells.

Although allogeneic cartilage transplantation has been performed against chondral defects, in which subchondral bone is maintained,^4^ it has not been well studied whether allogeneic cartilage in osteochondral defects, where transplants are exposed to abundant blood flow in the bone marrow, causes immune reactions. In this study, we found that transplanted *B2M^+/+^* cyiPSC-derived cartilage survived in osteochondral defects and was not immune rejected, but it did cause an accumulation of leukocytes including CD3^+^ T cells around them to some extent. The survival of allogeneic cyiPSC-derived cartilage in osteochondral defects despite the T cell accumulation can be accounted for by a previous study that reported chondrocytes express negative regulators of immune responses, including B7 family members, chondromodulin-I. and indoleamine 2,3-dioxygenase.^2^

On the contrary, transplanted *B2M^−/−^* cyiPSC-derived cartilage were surrounded by NKG2A^+^ NK cells. This result is consistent with previous finding that cells not expressing HLA class I molecules (HLA-ABC) on their surface elicit an innate immune response by NK cells.^28^ Following that work, recent studies have developed human iPSCs that lack HLA class I molecules but retain HLA-C^17^ or overexpress CD47^29^ to suppress the activation of NK cells. On the contrary, chondrocytes constitutively express LLT1, a ligand of inhibitory NKR-P1A NK cell receptor.^30^ These findings could account for our results, in which *B2M^−/−^* cyiPSC-derived cartilage was not rejected after allogeneic transplantation into osteochondral defects in monkey despite the accumulation of NK cells. These findings raise the hypothesis that cartilage derived from iPSCs lacking HLA class I molecules but retaining HLA-C^17^ or overexpressing CD47^29^ can evade accumulation of NK cells and serve as effective transplants for osteochondral defects. Further studies are needed to understand the immune reactions during the allogeneic transplantation of cartilage. The *B2M^−/−^* cyiPSCs- and *B2M^−/−^* cyiPSCs-derived chondrocytes/cartilage described in this study will contribute to such research.

## Data Availability

All original data are available upon request to the authors.
